# Supplementary material for: Analysis of chi angle distributions in free amino acids via multiplet fitting of proton scalar couplings
Source: Magn Reson (Gott). 2024 Aug 19;5(2):103–20. doi: 10.5194/mr-5-103-2024 (PMC11570886; doi:10.5194/mr-5-103-2024)
Supplement: The supplement related to this article is available online at: https://doi.org/10.5194/mr-5-103-2024-supplement. [file mr-5-103-2024-supplement.zip › Syed2024/data/fit1d_fitnmr_output.tar.gz!/fit1d/phe/500/bmse000045_simulation_1_nmredata/1H/resonances_1d.pdf]

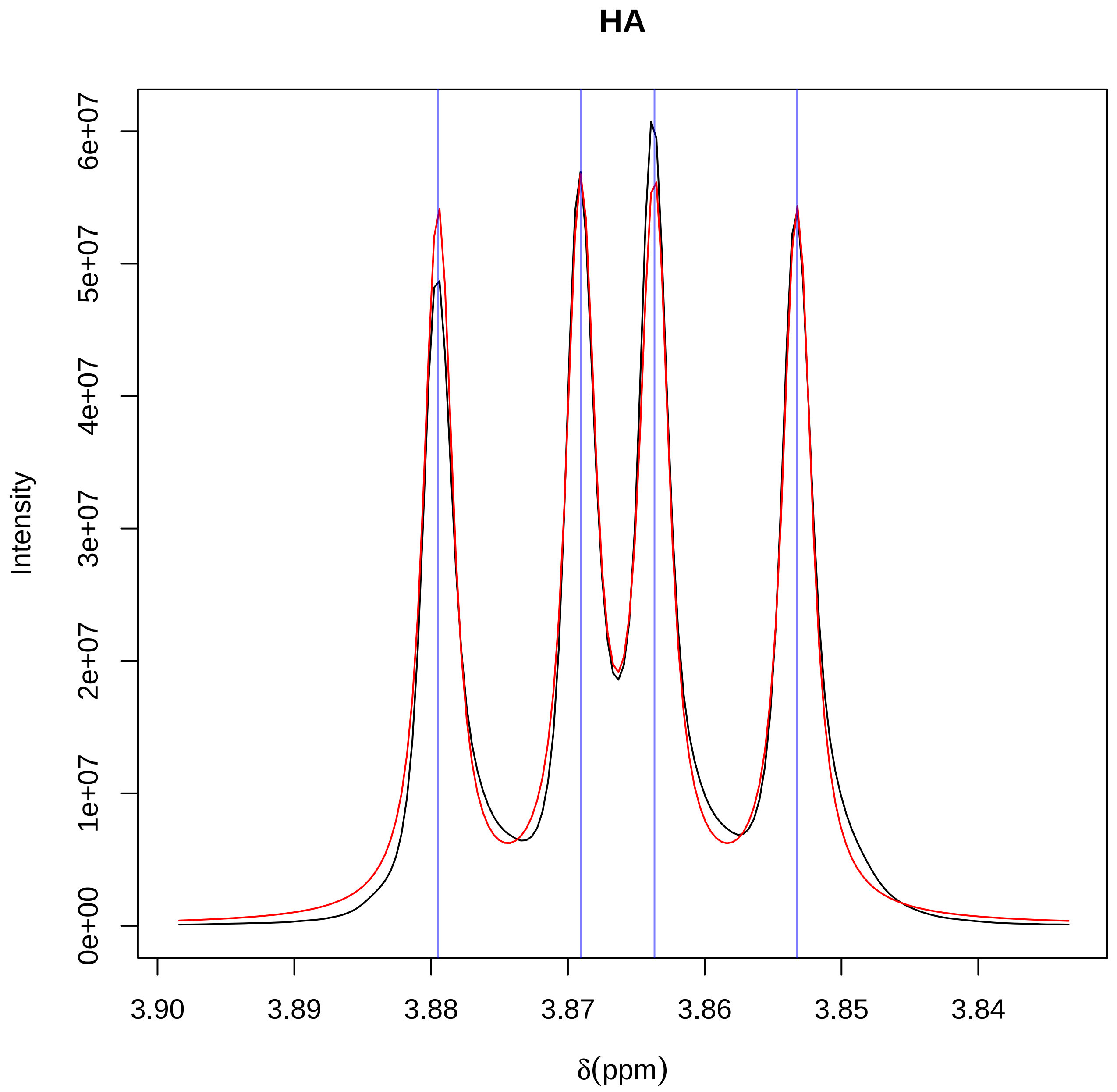

HA
Intensity
6e+07
5e+07
4e+07
3e+07
2e+07
1e+07
0e+00
3.90
3.89
3.88
3.87
3.86
3.85
3.84
δ(ppm)

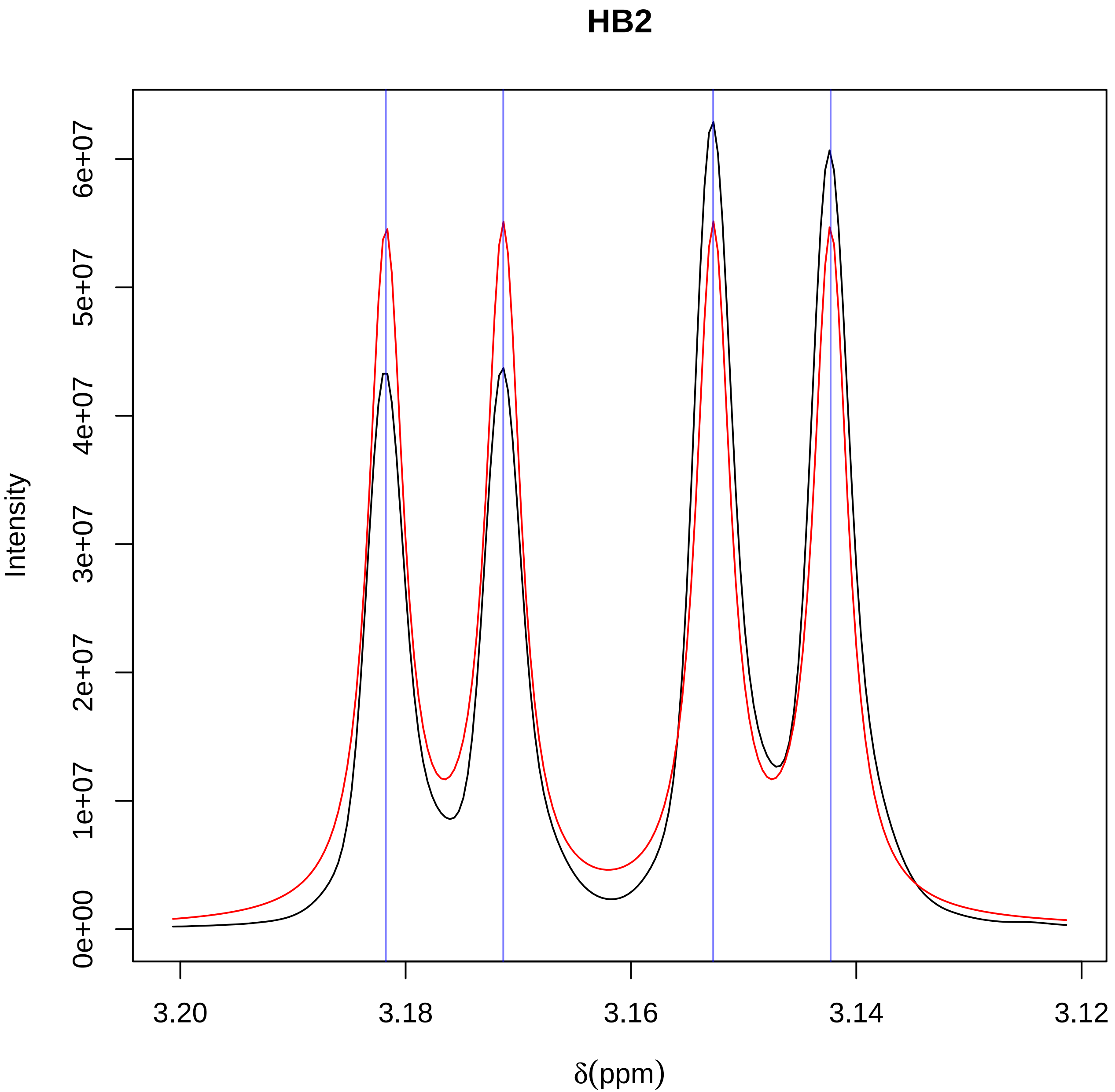
HB2
Intensity
0e+00
1e+07
2e+07
3e+07
4e+07
5e+07
6e+07
3.20
3.18
3.16
3.14
3.12
δ(ppm)

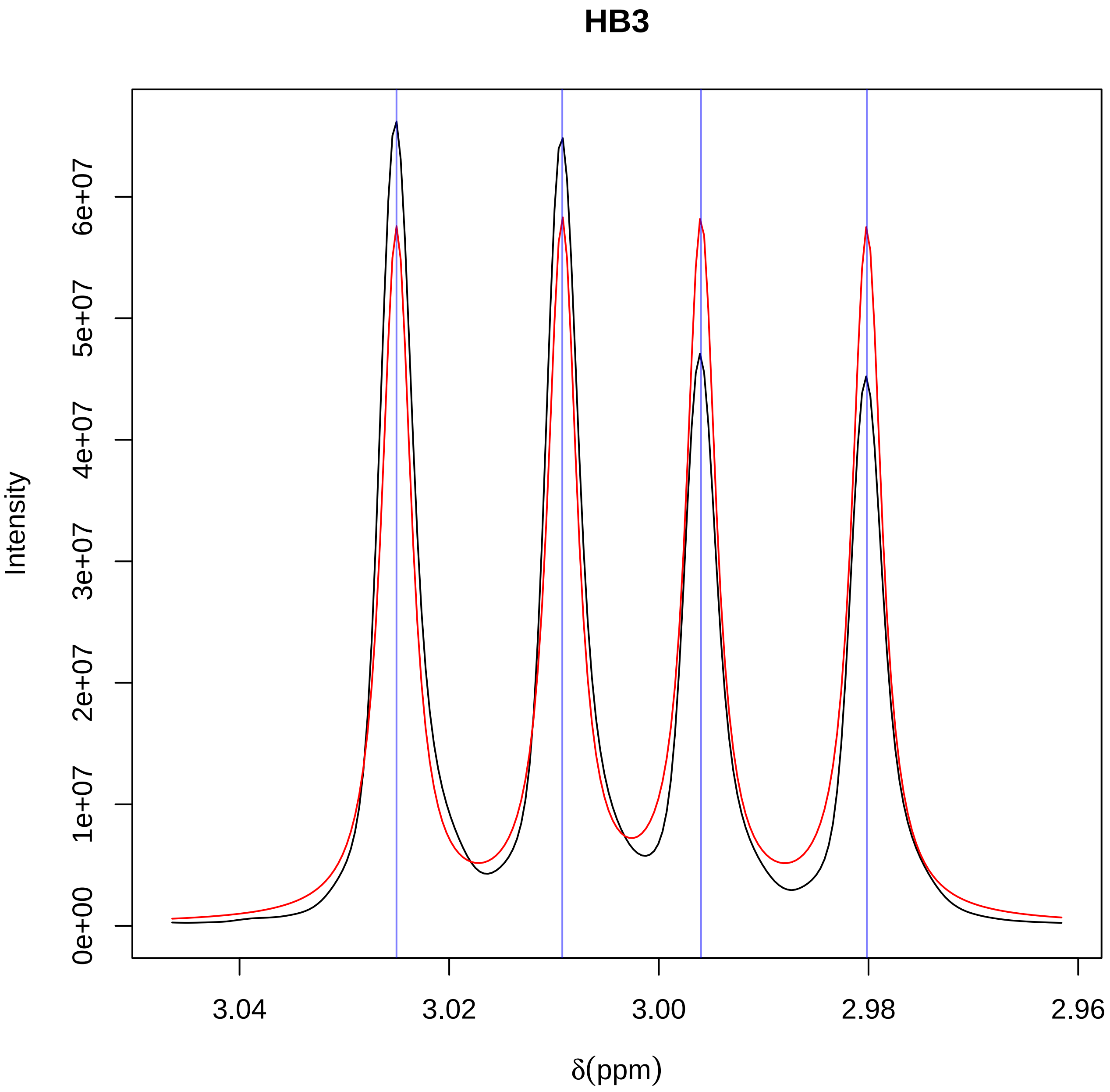
HB3
Intensity
0e+00
1e+07
2e+07
3e+07
4e+07
5e+07
6e+07
3.04
3.02
3.00
2.98
2.96
δ(ppm)

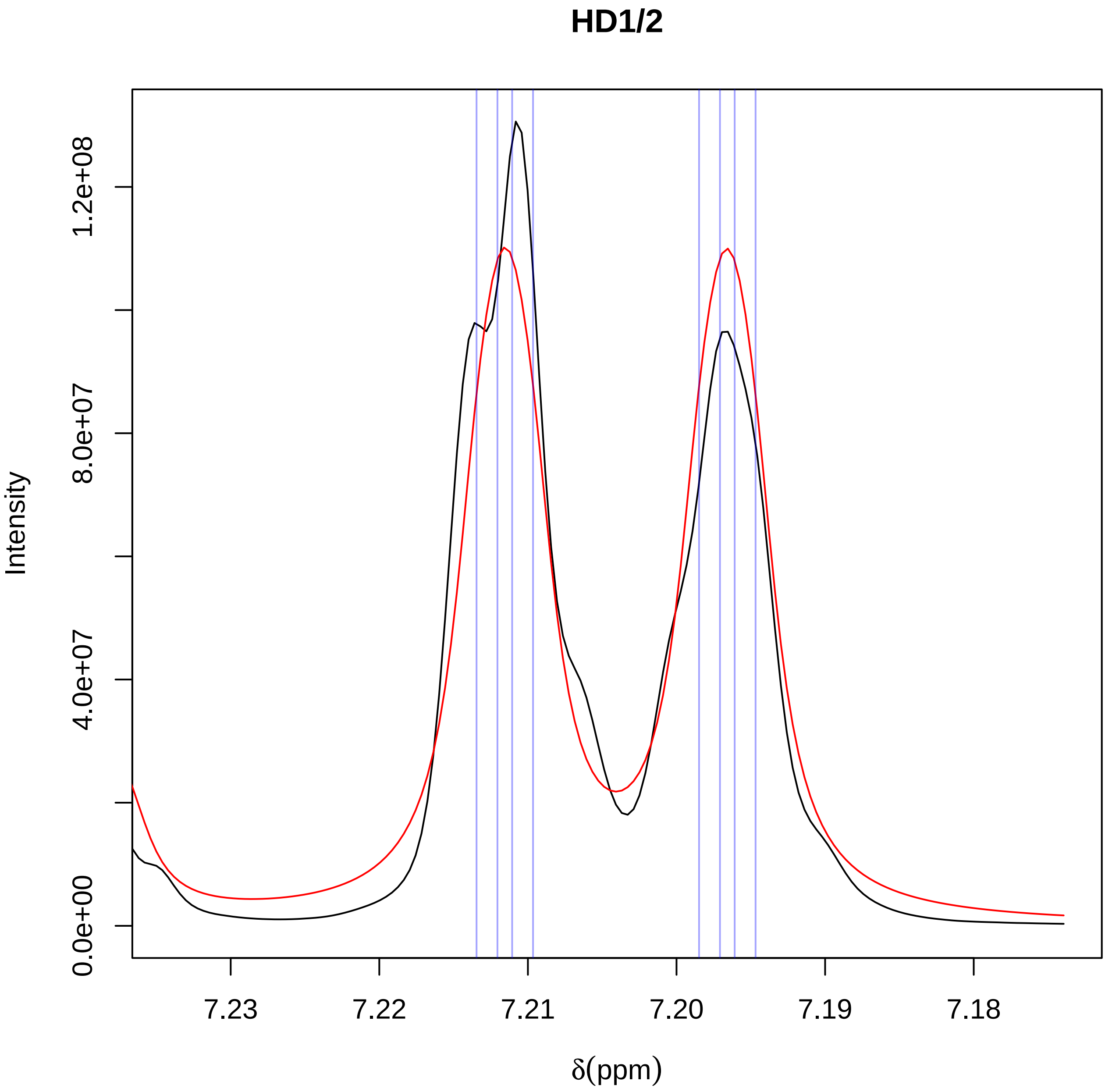
HD1/2
Intensity
0.0e+00
4.0e+07
8.0e+07
1.2e+08
7.23
7.22
7.21
7.20
7.19
7.18
δ(ppm)

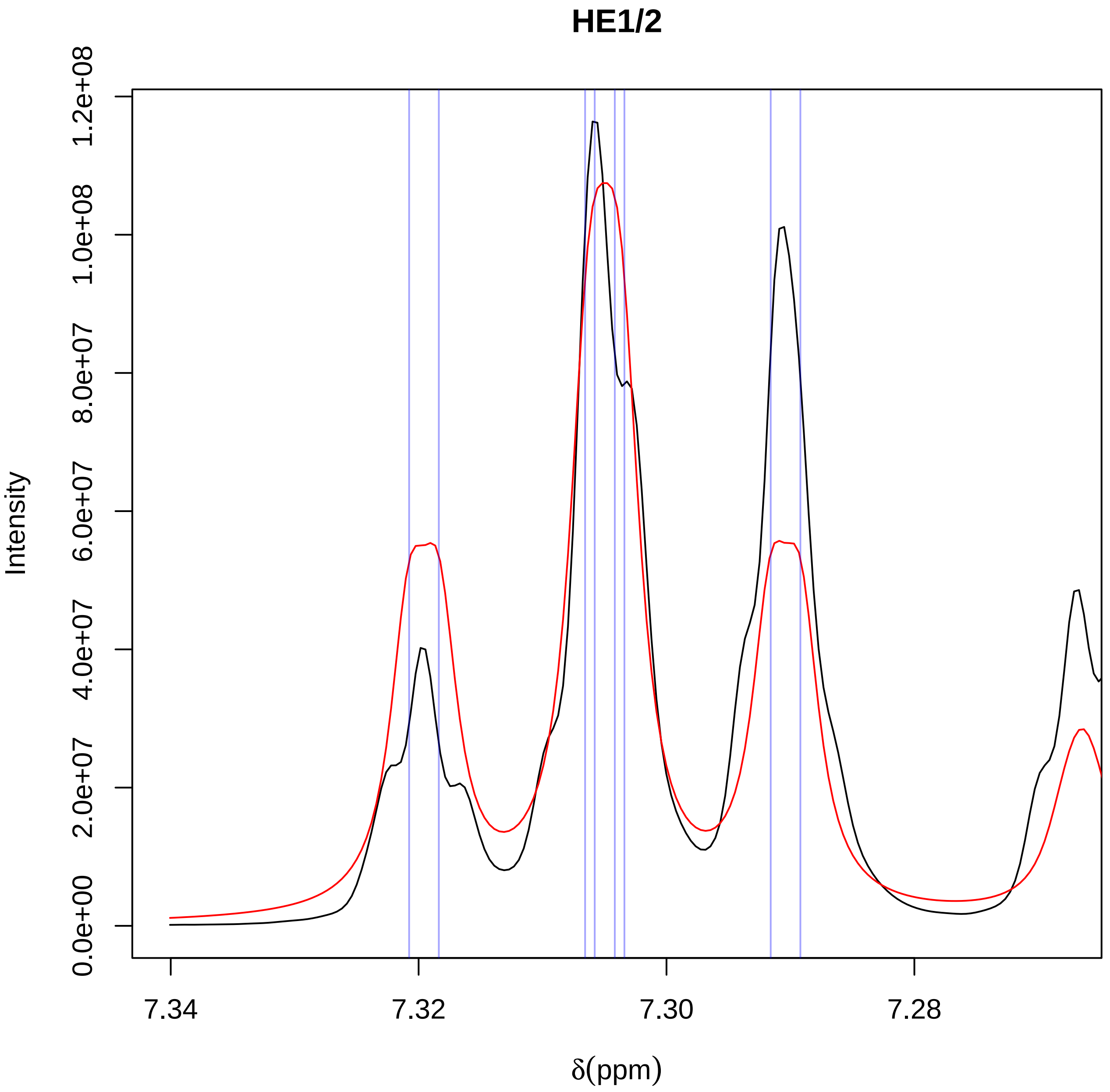

HE1/2
Intensity
0.0e+00
2.0e+07
4.0e+07
6.0e+07
8.0e+07
1.0e+08
1.2e+08
7.34
7.32
7.30
7.28
δ(ppm)

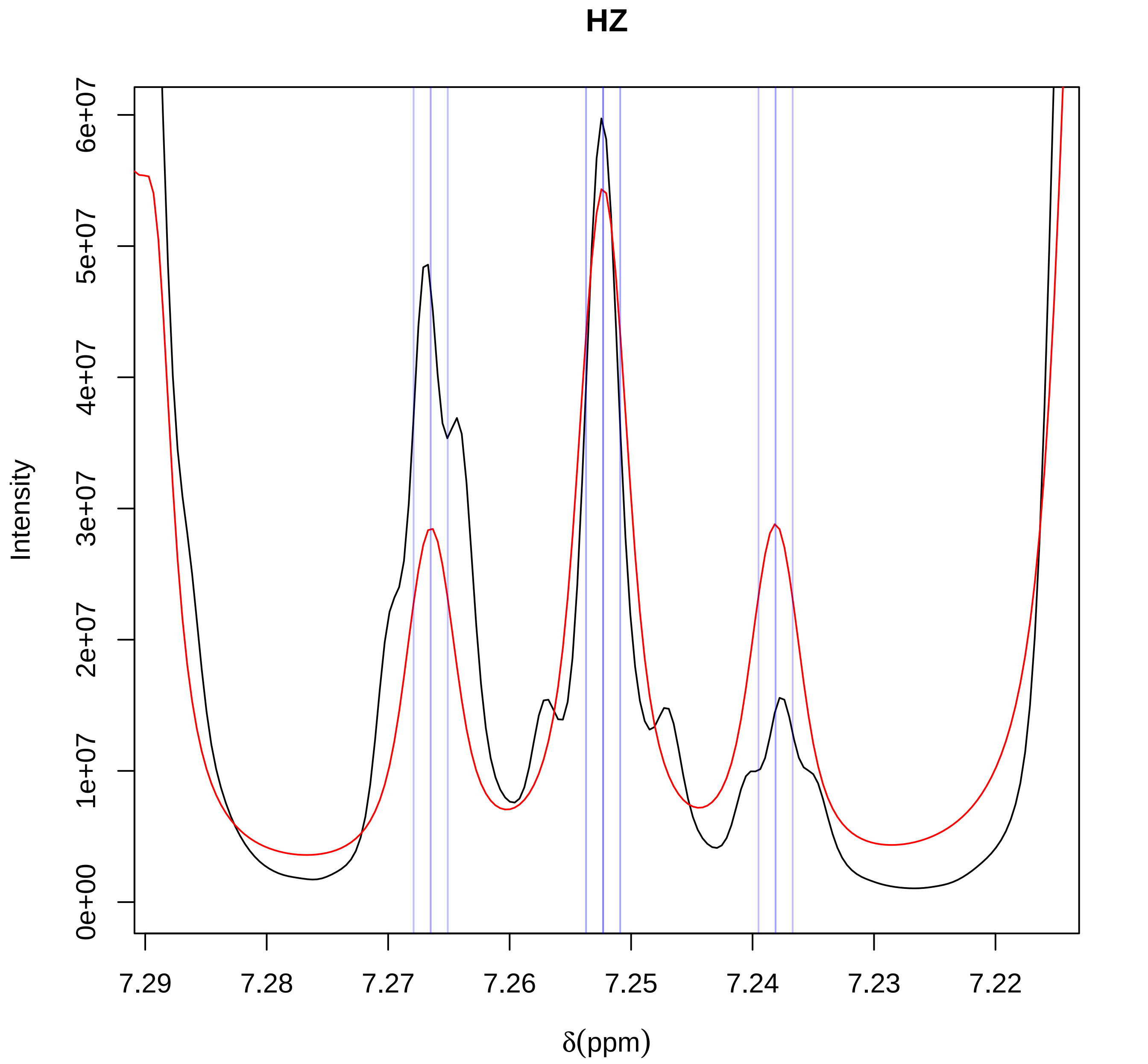
HZ
Intensity
6e+07
5e+07
4e+07
3e+07
2e+07
1e+07
0e+00
7.29
7.28
7.27
7.26
7.25
7.24
7.23
7.22
δ(ppm)
